# ACSL1, CH25H, GPCPD1, and PLA2G12A as the potential lipid-related diagnostic biomarkers of acute myocardial infarction

**DOI:** 10.18632/aging.204542

**Published:** 2023-02-24

**Authors:** Zheng-Yu Liu, Fen Liu, Yan Cao, Shao-Liang Peng, Hong-Wei Pan, Xiu-Qin Hong, Peng-Fei Zheng

**Affiliations:** 1 Department of Cardiology, Hunan Provincial People's Hospital, Changsha 410000, China; 2 Department of Epidemiology, Hunan Provincial People's Hospital (The First Affiliated Hospital of Hunan Normal University), Changsha 410000, China; 3 Clinical Medicine Research Center of Heart Failure of Hunan Province, Changsha 410000, China; 4 The First Affiliated Hospital of Hunan Normal University (Hunan Provincial People's Hospital), Changsha 410000, China; 5 Department of Emergency, Hunan Provincial People's Hospital, Changsha 410000, China; 6 Clinical Data Center, Hunan Provincial People's Hospital, Changsha 410000, China

**Keywords:** lipid, acute myocardial infarction, bioinformatics analysis, machine learning, gene

## Abstract

Lipid metabolism plays an essential role in the genesis and progress of acute myocardial infarction (AMI). Herein, we identified and verified latent lipid-related genes involved in AMI by bioinformatic analysis. Lipid-related differentially expressed genes (DEGs) involved in AMI were identified using the GSE66360 dataset from the Gene Expression Omnibus (GEO) database and R software packages. Gene ontology (GO) and Kyoto Encyclopedia of Genes and Genomes (KEGG) pathway enrichment analyses were conducted to analyze lipid-related DEGs. Lipid-related genes were identified by two machine learning techniques: least absolute shrinkage and selection operator (LASSO) regression and support vector machine recursive feature elimination (SVM-RFE). The receiver operating characteristic (ROC) curves were used to descript diagnostic accuracy. Furthermore, blood samples were collected from AMI patients and healthy individuals, and real-time quantitative polymerase chain reaction (RT-qPCR) was used to determine the RNA levels of four lipid-related DEGs. Fifty lipid-related DEGs were identified, 28 upregulated and 22 downregulated. Several enrichment terms related to lipid metabolism were found by GO and KEGG enrichment analyses. After LASSO and SVM-RFE screening, four genes (*ACSL1, CH25H, GPCPD1*, and *PLA2G12A*) were identified as potential diagnostic biomarkers for AMI. Moreover, the RT-qPCR analysis indicated that the expression levels of four DEGs in AMI patients and healthy individuals were consistent with bioinformatics analysis results. The validation of clinical samples suggested that 4 lipid-related DEGs are expected to be diagnostic markers for AMI and provide new targets for lipid therapy of AMI.

## INTRODUCTION

Acute myocardial infarction (AMI) has become the main cause of hospitalization and death worldwide, seriously threatening human health. Previous studies have suggested that AMI is a complex syndrome with multifactorial disorders. Its risk factors include early family history, smoking, hypertension, dyslipidemia, and diabetes [1–4]. The rupture of vulnerable and lipid-overloaded coronary atherosclerotic plaques can induce the formation of acute thrombus, leading to acute occlusion of blood vessels and progressing to AMI [5]. Dyslipidemia, especially elevated levels of low-density lipoprotein (LDL) cholesterol, is believed to play a key role in the pathogenesis of atherosclerosis [6, 7]. Atherogenesis begins when residues of LDL cholesterol, chylomicron, and very low-density lipoprotein (VLDL) cholesterol molecules enter the artery intima. Then, lipid radicals are oxidized and endocytosed by macrophages, followed by the formation of foam cells [6]. Previous studies have demonstrated that every 1% reduction in LDL cholesterol levels is associated with a 1% reduction in AMI risk [8, 9]. Currently, statin therapy has become the cornerstone for cholesterol-lowering drug therapy for coronary heart disease and AMI patients. Moreover, the combination of PCSK9 inhibitors with statins is recommended to reduce the risk of major adverse cardiovascular events (MACEs) in AMI patients [10]. However, the combination of lipid-lowering therapy still cannot eliminate the risk of MACEs in AMI patients. This might be partly because some lipid-related genes have not been identified. Therefore, identifying new lipid metabolism-related genes associated with AMI will help develop new lipid-lowering drugs to reduce the risk of MACEs in AMI patients.

Microarray analysis is an innovative and practical method to discern susceptibility genes to deal with coronary heart disease [11] and AMI [12]. Nevertheless, microarray analysis using differentially expressed genes (DEGs) might have limitations in reproducibility and sensitivity [13, 14]. Machine learning can enhance the prediction and accuracy of these key genes discerned using traditional microarrays or next-generation sequencing data [15]. The most frequently used machine learning techniques include the least absolute shrinkage and selection operator (LASSO) regression and support vector machine recursive feature elimination (SVM-RFE) algorithm [16]. Meanwhile, the combined application of LASSO regression and SVM-RFE algorithm in identifying new lipid-related genes involved in AMI has not been conducted. Therefore, in the present study, we analyzed the GSE66360 dataset from different perspectives: 1) DEGs among lipid-related AMI genes were identified using the “limma” R package and GO and KEGG pathway enrichment analyses. 2) Machine learning methods were applied to large-scale screening and diagnostic identification of AMI-related molecular markers. 3) Lipid-related gene expression levels screened by machine learning were validated using clinical cases.

## RESULTS

### Identification of lipid-related DEGs in GSE66360

**Figure 1:**
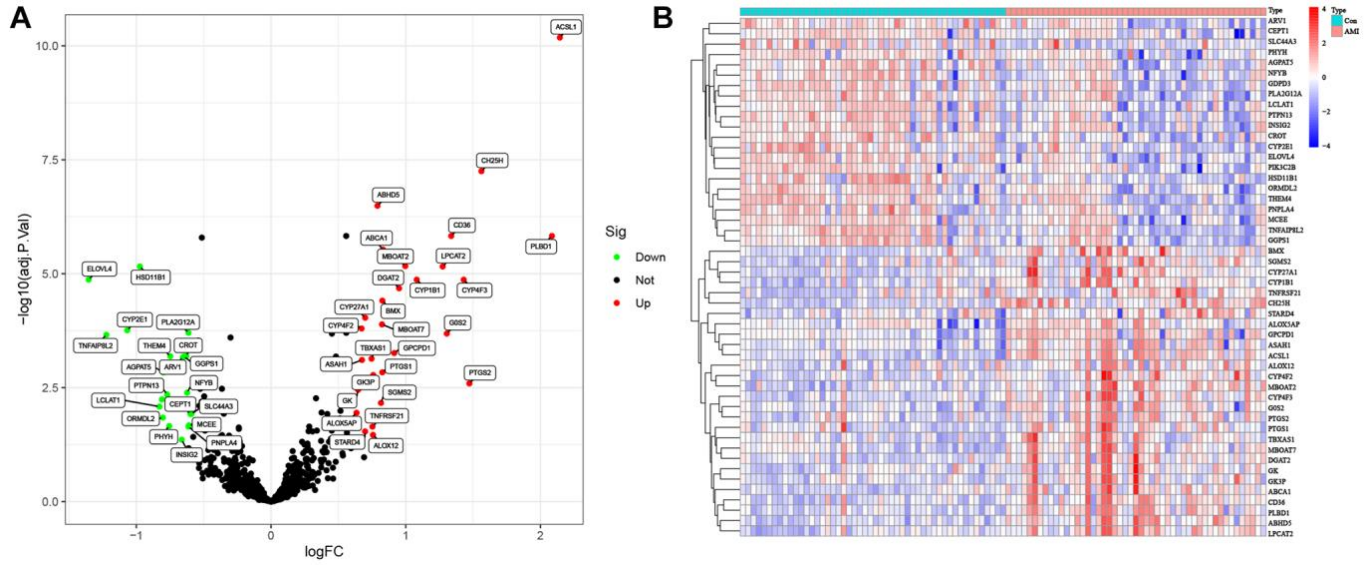
**Lipid-related differentially expressed genes (DEGs) in AMI and healthy samples.** (**A**) Volcano plot of the 673 lipid-related DEGs. Red dots represent significantly upregulated genes, and green significantly downregulated genes. (**B**) Heatmap of the 50 lipid-related DEGs in AMI and healthy samples.

**Figure 2:**
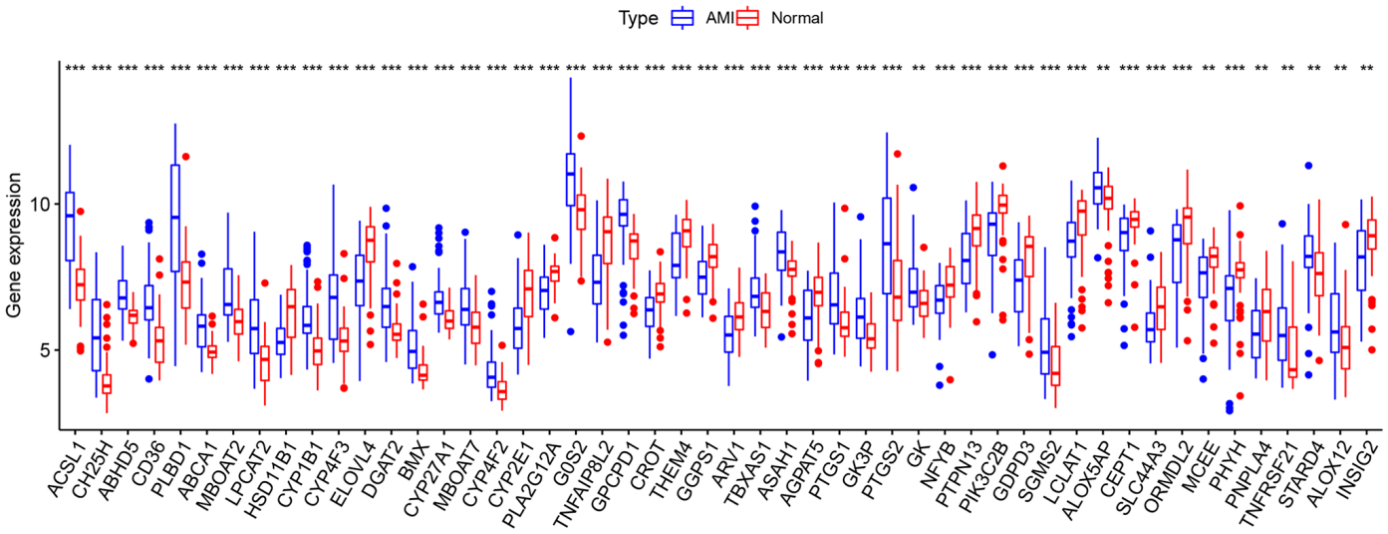
**Box plot of 50 lipid-related differentially expressed genes (DEGs) in AMI and healthy samples.**^ *^p < 0.05; ^**^p < 0.01; ^***^p < 0.005.

Due to the limitations of chip detection technology, a total of 673 lipid-related genes in 49 AMI samples and 50 normal samples were used to analyze DEGs. After data normalization and removal of batch differences, 50 lipid-related genes were identified, 28 upregulated and 22 downregulated (Table 1). These 50 lipid-related DEGs can be visualized in the volcano plot and heatmap (Figure 1A, 1B). The expression pattern between the two groups was highlighted based on the box plot (Figure 2). Among them, the top three upregulated genes were *ACSSL1* (Acyl-CoA Synthetase Long-Chain Family Member 1), *PLBD1* (Phospholipase B Domain Containing 1), and *CH25H* (Cholesterol 25-Hydroxylase). Meanwhile, the top three downregulated genes were *ELOVL4* (*ELOVL* Fatty Acid Elongase 4), *TNFAIP8L2* (*TNF* Alpha Induced Protein 8 Like 2), and *CYP2E1* (Cytochrome P450 Family 2 Subfamily E Member 1).

**Table 1:** The 50 differentially expressed lipid-related genes in AMI samples compared to healthy samples.

**Gene symbol**	**Log_2_FC**	**Changes**	***p*-value**	**Adjusted *p*-value**
ACSL1	2.1443758	Up	9.76E-14	6.57E-11
PLBD1	2.0855158	Up	1.33E-08	1.49E-06
CH25H	1.561894	Up	1.67E-10	5.63E-08
PTGS2	1.4727308	Up	1.64E-04	2.56E-03
CYP4F3	1.4307856	Up	2.77E-07	1.35E-05
CD36	1.3377248	Up	1.25E-08	1.49E-06
G0S2	1.3051173	Up	7.36E-06	2.06E-04
LPCAT2	1.274232	Up	1.13E-07	6.95E-06
CYP1B1	1.081695	Up	2.57E-07	1.35E-05
MBOAT2	0.996586	Up	9.09E-08	6.80E-06
DGAT2	0.9506635	Up	4.65E-07	2.09E-05
GPCPD1	0.9145163	Up	2.38E-05	5.52E-04
ABCA1	0.8308084	Up	3.50E-08	2.94E-06
BMX	0.8267136	Up	9.28E-07	3.90E-05
PTGS1	0.8263128	Up	8.69E-05	1.46E-03
MBOAT7	0.8240098	Up	3.66E-06	1.29E-04
SGMS2	0.8165743	Up	5.71E-04	6.86E-03
ABHD5	0.7901841	Up	1.44E-09	3.23E-07
GK3P	0.7584331	Up	1.02E-04	1.68E-03
ALOX12	0.7568656	Up	4.36E-03	3.45E-02
TNFRSF21	0.7529465	Up	2.53E-03	2.27E-02
TBXAS1	0.7461727	Up	3.82E-05	7.34E-04
CYP27A1	0.6990032	Up	2.48E-06	9.27E-05
STARD4	0.6983926	Up	3.56E-03	2.89E-02
ASAH1	0.6750605	Up	4.30E-05	7.82E-04
CYP4F2	0.6721891	Up	4.74E-06	1.59E-04
GK	0.6464259	Up	2.46E-04	3.52E-03
ALOX5AP	0.6346144	Up	1.04E-03	1.13E-02
SLC44A3	−0.590564	Down	1.18E-03	1.20E-02
CEPT1	−0.599105	Down	1.17E-03	1.20E-02
MCEE	−0.609681	Down	2.24E-03	2.12E-02
PLA2G12A	−0.612271	Down	6.79E-06	1.99E-04
PNPLA4	−0.612941	Down	2.48E-03	2.27E-02
NFYB	−0.623073	Down	2.92E-04	4.09E-03
CROT	−0.628778	Down	2.87E-05	6.43E-04
GGPS1	−0.647568	Down	3.19E-05	6.58E-04
ARV1	−0.654258	Down	3.44E-05	6.81E-04
INSIG2	−0.663348	Down	6.01E-03	4.40E-02
THEM4	−0.748033	Down	2.99E-05	6.49E-04
PHYH	−0.755787	Down	2.36E-03	2.21E-02
PIK3C2B	−0.764005	Down	4.21E-04	5.45E-03
PTPN13	−0.771322	Down	3.25E-04	4.47E-03
AGPAT5	−0.799176	Down	8.56E-05	1.46E-03
ORMDL2	−0.803203	Down	1.43E-03	1.44E-02
GDPD3	−0.810763	Down	4.53E-04	5.75E-03
LCLAT1	−0.828645	Down	7.23E-04	8.25E-03
HSD11B1	−0.973808	Down	1.14E-07	6.95E-06
CYP2E1	−1.067542	Down	5.45E-06	1.75E-04
TNFAIP8L2	−1.222639	Down	8.47E-06	2.19E-04
ELOVL4	−1.35438	Down	2.82E-07	1.35E-05

### Correlation analysis of lipid-related DEGs

**Figure 3:**
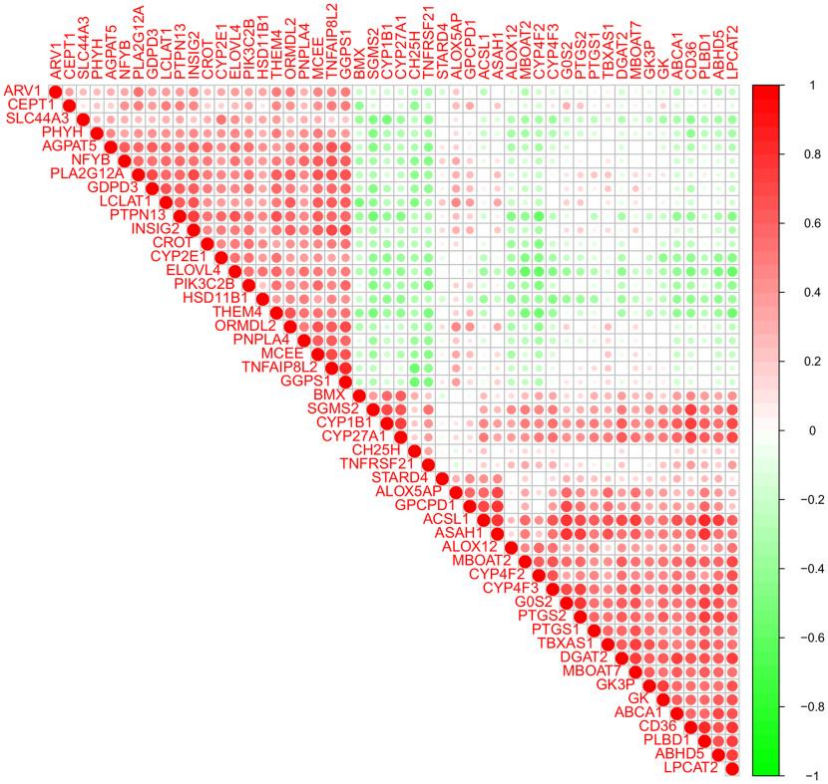
Pearson correlation analysis of the 50 lipid-related differentially expressed genes (DEGs).

The 50 lipid-related DEGs in the GSE66360 were significantly correlated by the Pearson correlation analysis (Figure 3). The positive correlation between the *PLBD1* and *ACSL1* was strongest; and the *CYP4F2* had the most obvious negative correlation with *PTPN13*. Moreover, the *ACSL1* was positively related to *GPCPD1* and *CH25H*; the *CH25H* was positively related to *GPCPD1* and *ACSL1*; the *PLA2G12A* was positively related to *GPCPD1* while negatively related to *CH25H*.

### Functional analyses of lipid-related DEGs

**Figure 4:**
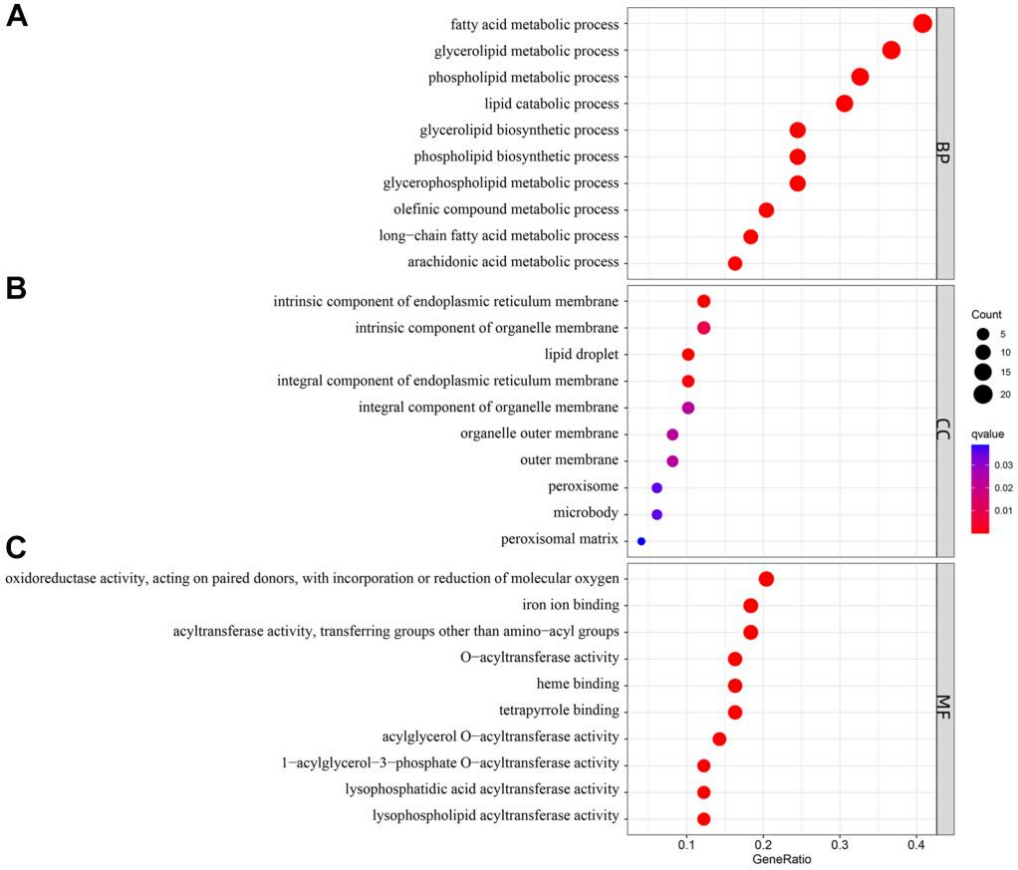
**Gene Ontology (GO) enrichment analysis of 50 differentially expressed genes (DEGs).** Abbreviations: (**A**) BP: biological process; (**B**) CC: cellular component; (**C**) MF: molecular function.

**Figure 5:**
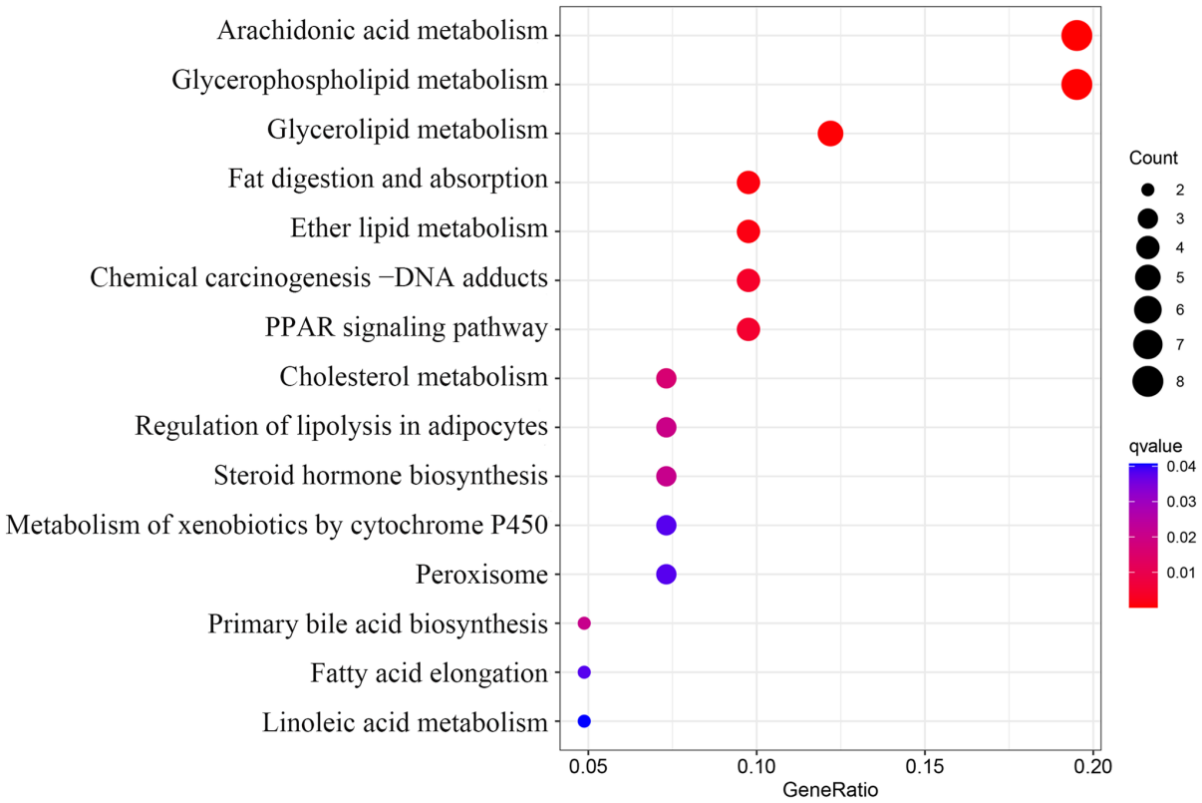
Kyoto Encyclopedia of Genes and Genomes (KEGG) pathway analysis of 50 lipid-related differentially expressed genes (DEGs).

The biological functions of lipid-related DEGs were determined by GO and KEGG enrichment analyses using R software. The most enriched GO terms were fatty acid metabolic, glycerolipid metabolic, and phospholipid metabolic processes (biological processes, Figure 4A); intrinsic component of endoplasmic reticulum membrane, integral component of endoplasmic reticulum membrane, and lipid droplet (cellular components, Figure 4B); oxidoreductase activity, iron ion binding, and acyltransferase activity (molecular functions, Figure 4C). The KEGG enrichment analyses showed that lipid-related DEGs were involved in the arachidonic acid metabolism, glycerophospholipid metabolism, chemical carcinogenesis-DNA adducts, and the PPAR signaling pathway (Figure 5). The details of these analyses can also be found in Supplementary Table 1.

### Screening key DEGs by LASSO logistic regression and SVM-RFE

**Figure 6:**
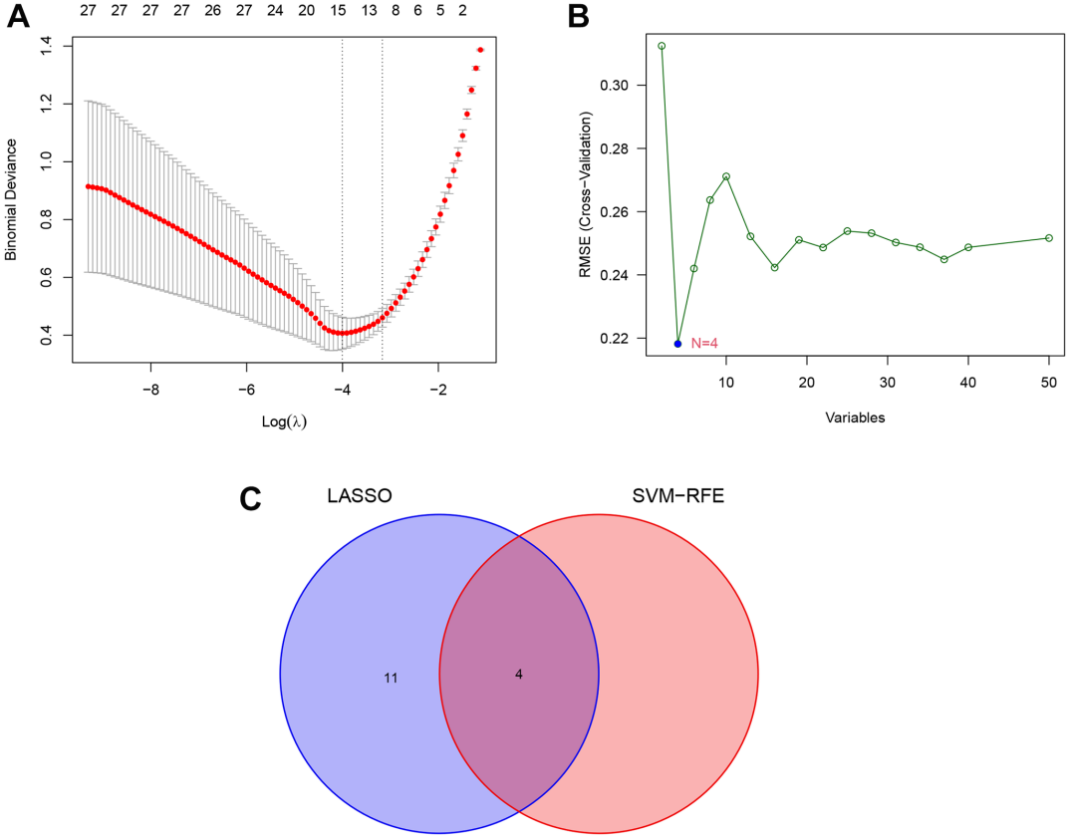
**Identification of key lipid-related DEGs by machine learning methods.** (**A**) Least absolute shrinkage and selection operator (LASSO) logistic regression screening of key lipid-related DEGs. (**B**) Support vector machine-recursive feature elimination (SVM-RFE) algorithm screening of key lipid-related DEGs. (**C**) Venn diagram of the intersection of diagnostic markers obtained by the two algorithms.

The LASSO logistic regression identified 15 lipid-related genes based on the optimum λ value (Figure 6A), whereas the SVM-RFE algorithm identified four genes (Figure 6B). Four overlap genes including *ACSL1*, *CH25H*, *GPCPD1* (Glycerophosphocholine Phosphodiesterase 1), and *PLA2G12A* (Phospholipase A2 Group XIIA) were identified by LASSO and SVM-RFE algorithm as key lipid-related DEGs for subsequent analysis (Figure 6C). In addition, other 14 genes identified by LASSO regression are listed in Supplementary Table 2.

### ROC curves between AMI and control groups

**Figure 7:**
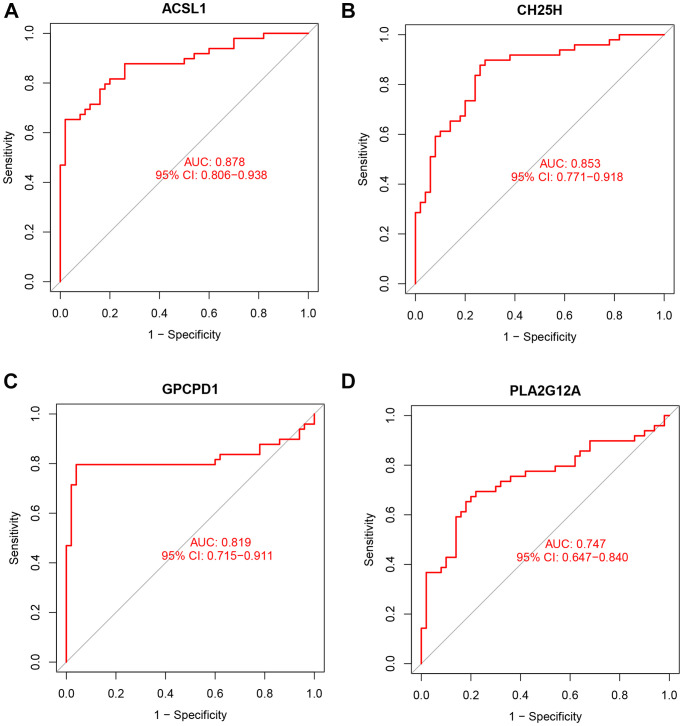
**ROC curve analysis.** ROC curve of *ACSL1* (**A**), *CH25H* (**B**), *GPCPD1* (**C**), *PLA2G12A* (**D**) in GSE66360 dataset.

The expression of the four lipid-related DEGs between the AMI and control samples of the GSE66360 dataset were analyzed using R software, and the ROC curves were constructed. The area under the curve (AUC), unified with specificity and sensitivity, verified the intrinsic validity of diagnostic tests [17]. Four lipid-related DEGs had a superior diagnostic value for AMI. Among them, the gene with the most significant diagnostic value was *ACSL1* (AUC = 0.878). The other genes are: *CH25H* (AUC = 0.853), *GPCPD1* (AUC = 0.819), and *PLA2G12A* (AUC = 0.747) (Figure 7). These four lipid-related DEGs can be considered underlying diagnostic biomarkers for AMI.

### Validation by RT-qPCR

**Figure 8:**
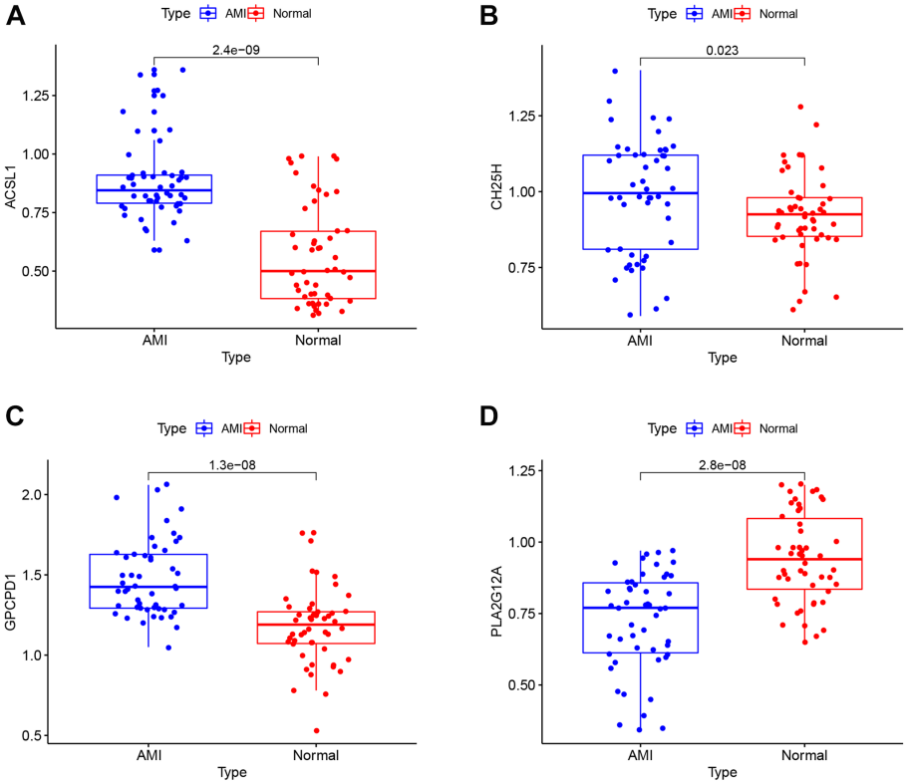
**RT-qPCR analysis.** The mRNA expression levels of *ACSL1* (**A**), *CH25H* (**B**), *GPCPD1* (**C**), *PLA2G12A* (**D**) in clinical samples.

To verify the dependability of the GSE66360 dataset, RT-qPCR was used to validate the expression levels of the above four lipid-related genes in clinical samples. The clinical data of AMI and control groups are summarized in Table 2. The RT-qPCR results suggested in Figure 8, that the expression level of *PLA2G12A* (*p* = 2.8e-08) increased in controls compared to the AMI group, while *ACSL1* (*p* = 2.4e-09), *CH25H* (*p* = 0.023), and *GPCPD1* (*p* = 1.3e-08) were higher in AMI group. Hence, the RT-qPCR results performed were consistent with the main bioinformatic analysis.

**Table 2:** Clinical characteristic between control and AMI group.

**Characteristic**	**Control (*n* = 50)**	**AMI (*n* = 50)**	***p***
Male [*n* (%)]^a^	35 (70.0)	34 (68.0)	0.829
Age (years)^a^	55.16 ± 7.22	56.52 ± 6.79	0.673
BMI (kg/m²)^a^	20.98 ± 2.74	23.69 ± 3.20	0.305
Smoking [*n* (%)]^b^	11 (22.0)	23 (46.0)	0.011
Alcohol [*n* (%)]^b^	13 (26.0)	21 (42.0)	0.091
Hypertension [*n* (%)]^b^	15 (30.0)	30 (60.0)	0.003
Type 2 Diabetes [*n* (%)]^b^	10 (20.0)	19 (38.0)	0.047
CK (U/L)^a^	125.84 ± 9.35	1162.1 ± 576.30	6.20E-9
CK-MB (U/L)^a^	15.5 ± 3.06	130.35 ± 19.76	1.14E-11
ApoA1 (g/L)^a^	1.38 ± 0.30	0.96 ± 0.19	0.045
ApoB (g/L)^a^	0.82 ± 0.19	1.01 ± 0.29	0.037
TC (mmol/L)^a^	3.96 ± 0.80	4.12 ± 1.14	0.029
TG (mmol/L)^a^	1.29 ± 0.41	1.50 ± 0.66	0.023
HDL-C (mmol/L)^a^	1.63 ± 0.47	1.10 ± 0.29	0.030
LDL-C (mmol/L)^a^	2.25 ± 0.94	3.57 ± 1.26	0.003
Creatinine (μmol/L)^a^	86.14 ± 13.07	88.98 ± 17.49	0.039
Troponin T (μg/L)^a^	0.04 ± 0.03	3.22 ± 1.19	2.87E-5

Abbreviations: BMI: Body mass index; HDL-C: high-density lipoprotein cholesterol; LDL-C: low-density lipoprotein cholesterol; Apo: Apolipoprotein; TC: Total cholesterol; TG: Triglyceride; CK: creatine kinase; CK-MB: creatine kinase-myocardial band. ^a^Mean ± SD determined by *t*-test. ^b^The rate or constituent ratio between the different groups was analyzed by the χ^2^ test.

### Verification of the potential biomarkers for AMI

**Figure 9:**
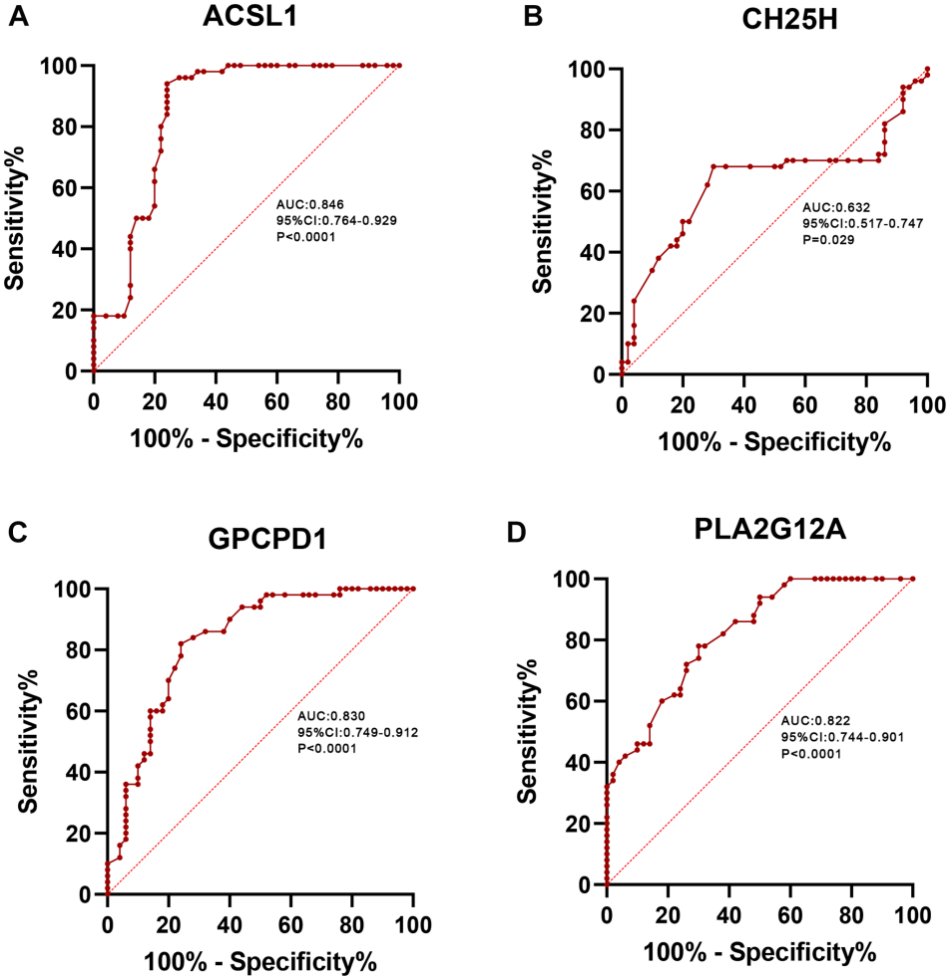
**ROC curve analysis.** ROC curve of *ACSL1* (**A**), *CH25H* (**B**), *GPCPD1* (**C**), *PLA2G12A* (**D**) in clinical samples.

Furthermore, we analyzed the gene expression levels in the AMI group and healthy individuals using the ROC curve to verify the diagnostic value of the four screened lipid-related genes as shown in Figure 9. The AUC values of *ACSL1*, *CH25H*, *GPCPD1* and *PLA2G12A* were 0.846 [95% confidence interval (CI): 0.764–0.929], 0.632 (95% CI: 0.517–0.747), 0.830 (95% CI: 0.749–0.912), and 0.822 (95% CI: 0.744–0.901), respectively. This result showed that these lipid-related DEGs are diagnostic biomarkers for AMI.

## DISCUSSION

Acute myocardial infarction (AMI) refers to hypoxia caused by coronary atherosclerosis stenosis and myocardial necrosis caused by acute and persistent ischemia [18]. Dyslipidemia is a known risk factor for AMI [19], and lipid-lowering therapy is the treatment cornerstone. Several convincing studies have shown that the combined effect of lowering triglyceride, LDL cholesterol, and total cholesterol levels yield higher cardiovascular risk than lowering LDL cholesterol levels alone [20–22]. The accumulated molecular genetic data indicate that many genes are related to AMI occurrence, including lipid-related genes [23]. However, the lipid-related genes involved in AMI have not been completely identified. Thus, it is necessary to comprehend the role of lipid-related genes in AMI diagnosis and treatment.

Herein, we retrieved data of AMI patients (GSE66360) and subjected it to differential genes analysis, and identified lipid-related DEGs associated with AMI. Lipid-related DEGs were then subjected to GO and KEGG enrichment analyses. LASSO regression is a machine learning method that recognizes variables by looking for a λ value for a minimal classification error [24]. SVM-RFE is another machine learning method that finds optimal variables through subtracting SVM-generated feature vectors [25]. We used these two algorithms to screen characteristic variables and created an optimal classification model. Four lipid-related genes *(ASCL1*, *CH25H*, *GPCPD1*, and *PLA2G12A*) were identified based on these two methods, which significantly impact AMI diagnosis. Moreover, the findings of *CH25H* were controversial compared with previous studies and should be interpreted with caution. Nevertheless, the *p* values of these four lipid-related genes were < 0.05, verified by RT-qPCR and consistent with our bioinformatic analysis results.

*ACSL1* is a key rate-limiting enzyme in lipid metabolism [26], catalyzing the energy production of fatty acids or the production of phospholipids, cholesterol esters, and triglycerides [27]. Previous studies have shown that heart-specific overexpression of *ACSL1* in mice increases triglyceride accumulation in cardiomyocytes [28]. Li et al. demonstrated that inhibiting *ACSL1* expression in the heart can reduce lipid metabolism and promote the regeneration of cardiomyocytes [29]. A cohort study has shown that the expression level of *ACSL1* in peripheral blood leukocytes of AMI patients was higher than that of healthy controls, and this high expression was a risk factor for AMI [30]. A recent study confirmed that the overexpression of *ACSL1* can reduce fatty acid β-oxidation and increase plasma triglyceride levels by regulating the *PPARγ* pathway, which is one of the mechanisms that can promote the pathogenesis of AMI [31]. These results supported the findings of our bioinformatic analysis and suggested that *ACSL1* plays a pathological role in AMI through lipid metabolism and might be a promising AMI biomarker. Moreover, *PLA2G12A* is a secreted phospholipase A2, but its physiological function is largely unclear. In humans, there is a suggestive association between a *PLA2G12A* polymorphism and response to anti-vascular endothelial growth factor therapy in patients with exudative age-related macular degeneration [32]. Alexandros et al. showed that *PLA2G12A* is highly expressed in aortic endothelial cells *in vivo* and may inhibit atherosclerosis by reducing the adhesion properties of vascular endothelial cells, which confirmed *PLA2G12A* as a candidate gene for atherosclerosis protection [33]. This was consistent with our findings that *PLA2G12A* was downregulated in AMI samples and was a protective gene, possibly by reducing vascular adhesion to decrease AMI incidence.

*CH25H* regulates cholesterol and lipid metabolism by converting cholesterol to 25-HC, and plays an important role in regulating cellular inflammatory states and cholesterol biosynthesis in endothelial cells and monocytes [34]. *CH25H* and 25-HC were traditionally regarded as key regulators to maintain cholesterol homeostasis by inhibiting sterol regulator-binding protein (SREBP) and liver X receptor (LXR) [35]. Elizabeth et al. showed that 25-HC production promotes the formation of macrophage foam cells and increases susceptibility to atherosclerosis, thereby increasing AMI risk [36]. However, the pro-inflammatory role of *CH25H* in atherosclerosis remains controversial. Other studies have shown that *CH25H* is involved in macrophages’ functional endothelium and anti-inflammatory phenotype and that *CH25H* ablation increases susceptibility to atherosclerosis [37]. Our current study suggested that *CH25H* was upregulated in AMI samples, consistent with the Elizabeth et al. results. This contradiction might be partly due to different experimental conditions requiring further study. Additionally, GPCPD1 is a key enzyme in choline and phospholipid metabolism. GPCPD1 has also been reported to be involved in the complex network of enzymatic reactions regulating choline metabolism [38]. It can cleave glycerophosphocholine to form glycerol-3-phosphate and choline [39]. GPCPD1 has been reported to promote cell migration, metastasis, adhesion, and diffusion in breast, endometrial, and ovarian cancers. However, its biological role in cardiovascular disease remains unclear. Hence, more studies are needed to further verify our current findings.

In the current research, several correlations between four key lipid-related DEGs were also noticed. Correlation analysis indicated that the *ACSL1*, *CH25H*, *GPCPD1*, and *PLA2G12A* genes may influence the occurrence of AMI by synergistically regulating the same lipid metabolic pathway. Meanwhile, the functional analyses were also performed to evaluate the potential biological functions of lipid-related DEGs. The GO enrichment analysis showed that these genes were closely related to fatty acid metabolism. Furthermore, the KEGG enrichment analysis revealed that the lipid-related genes were primarily associated with the *PPAR* signaling pathway. *PPAR* is activated by fatty acids and their derivatives, thereby creating a lipid signaling network between the cell surface and the nucleus [40]. As lipid sensors and master regulators, *PPAR* controls the expression of genes that function in lipid metabolism [41]. The *PPAR* signaling pathway, a crossing regulator of lipid signaling and inflammation, [40] was enriched, indicating that it plays a crucial role in lipid metabolism response to AMI. A previous study has found that the downregulation of *PPARγ* contributes to the activation and aggregation, eventually forming micro-thromboses, finally leading to myocardial dysfunction [42]. These results indicated that these lipid-related genes might affect AMI occurrence through the *PPAR* signaling pathway. However, further research is required to confirm the correlations between these key genes.

However, our current study also has some limitations. First, we used the dataset from circulating endothelial cells to perform the bioinformatics analysis, and used the peripheral blood mononuclear cells from myocardial infarction and normal people for verification. Although there was some sample heterogeneity, our research, like other studies using GSE66360 dataset [43–45], obtained a satisfactory result, which fully supported our conclusion. However, more studies are needed to further confirm our findings. Second, the included clinical samples were relatively small. Therefore, our conclusions must be verified by a larger AMI cohort. Third, lipid-related DEGs were only confirmed in clinical samples, and their potential functions were not demonstrated in AMI cells or animal models. Hence, more *in vivo* and *in vitro* studies are needed to clarify the underlying mechanisms of these key genes in AMI.

In summary, four lipid-related genes involved in AMI were confirmed by bioinformatics analysis and machine learning methods. These genes might influence AMI occurrence by regulating lipid metabolism. Our current findings might help understand the mechanisms of lipid metabolism-related genes in AMI and develop future lipid-lowering treatment strategies for AMI.

## MATERIALS AND METHODS

### Lipid-related gene dataset

The workflow diagram of this research was shown in Supplementary Figure 1. A total of 742 lipid-related genes were retrieved from Gene Set Enrichment Analysis (https://www.gsea-msigdb.org/gsea/index.jsp) (Supplementary Table 3). The AMI dataset (GSE66360) was downloaded from the GEO website (https://www.ncbi.nlm.nih.gov/geo/). The GSE66360 dataset including a total of 99 circulating endothelial cell samples that collected from 49 AMI and 50 control subjects, and this dataset was based on the GPL570 platform (Affymetrix Human Genome U133 Plus 2.0 Array). A total of 21629 genes were detected in the GSE66360 dataset.

### Identification of lipid-related DEGs

The “limma” R package was used to identify lipid-related DEGs between AMI patients and normal participants. The threshold values were *p* < 0.05 and |log fold change (FC)| > 0.585 [46–48]. Heatmaps, volcano plots, and boxplot charts were plotted using “heatmap” and “ggplot2” R packages.

### Functional enrichment analysis of lipid-related genes

The GO and KEGG pathway enrichment analyses were conducted using the “enrichplot” R package. Cell composition, biological processes, and molecular functions were included in the GO analysis.

### Screening of lipid-related genes through SVM-RFE and LASSO logistic regression

The “glmnet” R package was used to perform LASSO logistic regression which the response type set as binomial and alpha set as 1 to identify lipid-related genes [49]. LASSO regression is a regularized penalty regression method, combining ridge regression and subset selection. It applies ordinary least squares, but the sum of absolute values of the regression coefficients is less than the predetermined constant value [50]. LASSO logistic regression is a generalization of the binomial distribution of the LASSO output variable. Herein, we used LASSO to screen lipid-related genes. Moreover, SVM-RFE is a machine learning method based on support vector machines that identify optimal variables by removing SVM-generated feature vectors [51], and the thresholds were set as follows: halve.above = 100 and k = 5. The “E1071” R package was used to establish the SVM module to sift lipid-related genes. Then, the intersections of lipid-related genes sifted by LASSO and SVM-RFE were applied to AMI diagnostic analysis, and the ROC curves were plotted.

### Clinical validation samples

From September 2021 to May 2022, 50 AMI patients (AMI group) and healthy participants (control group) were recruited from the Hunan Provincial People’s Hospital. The blood samples were collected from AMI patients within hours of admission with chest pain and before using antiplatelet or anticoagulant to eliminate the influence of possible changes in blood status after pharmacological intervention. All AMI patients underwent percutaneous coronary intervention (PCI) within 12 h of the chest pain onset. The AMI patients were diagnosed based on the 2018 guidelines for diagnosing AMI patients [52]. A total of 50 healthy individuals were enrolled in the hospital physical examination center in the same period. The exclusion criteria were: (i) active inflammation; (ii) patients receiving thrombolysis and with other underlying heart diseases (e.g., severe valvular abnormalities, cardiomyopathy, or congenital heart disease); and (iii) patients who had hepatic and/or renal dysfunction, tumors, and autoimmune diseases. All participants provided written informed consent before the beginning of the study. This research was approved by the Ethics Committee of the Hunan Provincial People’s Hospital (approval number: [2021]-41).

### Real-time quantitative polymerase chain reaction (RT-qPCR)

Peripheral blood was obtained from blood samples of patients using RNeasy™ Mini Kit (QIAGEN, Frankfurt, Germany) to extract total RNA. Total RNA was reverse transcribed into cDNA using the PrimeScript RT reagent kit (Takara Bio, Japan). RT-qPCR was performed with a LightCycler 480 II Real-time PCR instrument (Roche, Switzerland) using the TransStart Top Green qPCR SuperMix (AQ131-03, Transgen, Beijing, China).

### Statistical analysis

All bioinformatics and Pearson’s correlation analyses were performed using R software (version 4.6.0, http://www.R-project.org). SPSS software (version 22.0) was used to analyze clinical data. Clinical characteristic data were analyzed using Student’s *t*-test and χ^2^ test. R and Grap Pad Prism software were used for ROC curve analysis. A *p* < 0.05 was considered statistically significant.

## Supplementary Materials

Supplementary Figure 1

Supplementary Table 1

Supplementary Table 2

Supplementary Table 3
