# Supplementary figures and images for: ACSL1, CH25H, GPCPD1, and PLA2G12A as the potential lipid-related diagnostic biomarkers of acute myocardial infarction
Source: Aging (Albany NY). 2023 Feb 24;15(5):1394–411. doi: 10.18632/aging.204542 (PMC10042701; doi:10.18632/aging.204542)

## SUPPLEMENTARY FIGURE

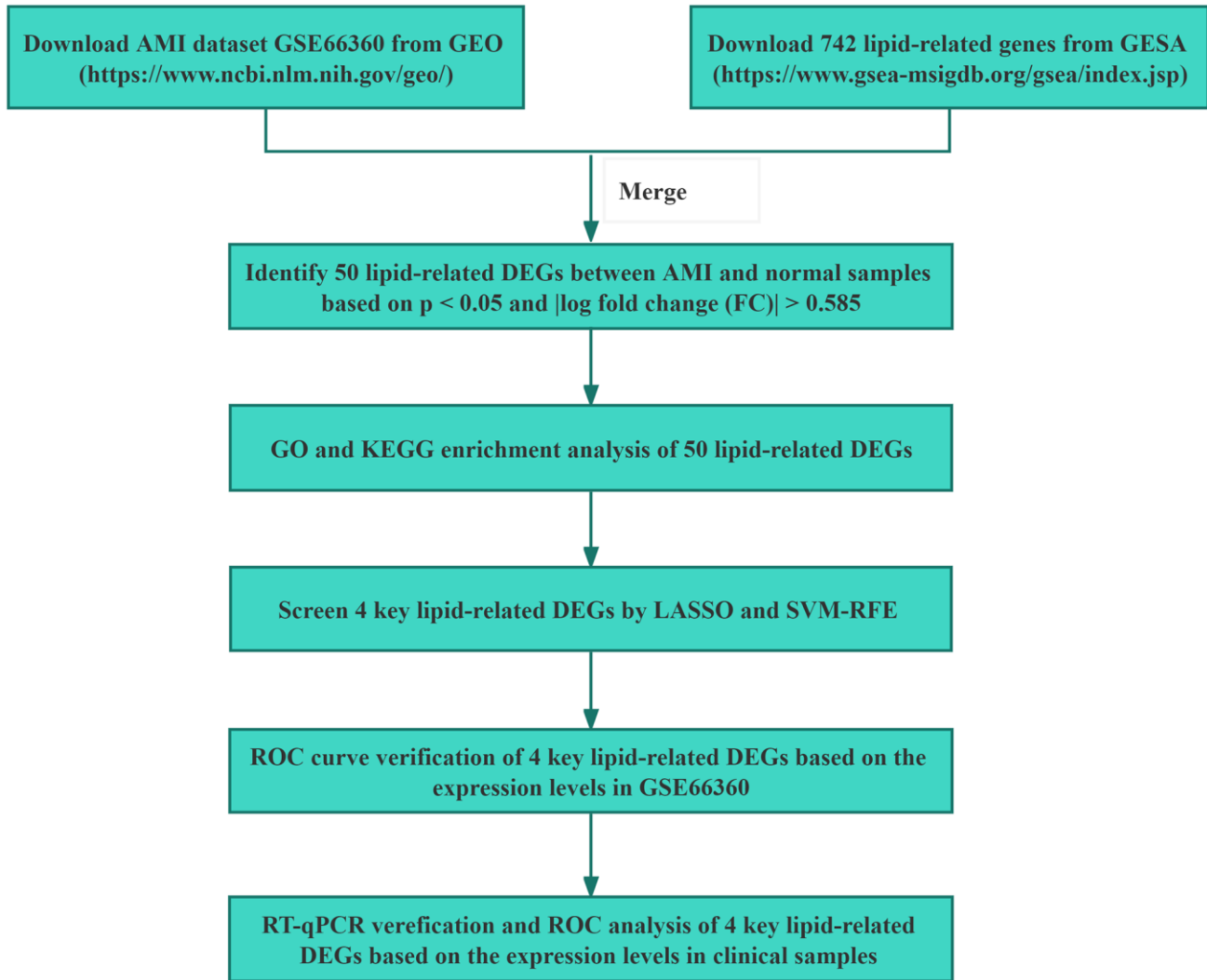

Supplementary Figure 1. A flow chart for analysis.

Supplement: Supplementary Figure 1 [file aging-15-204542-s001.pdf]
