# Supplementary material for: ACSL1, CH25H, GPCPD1, and PLA2G12A as the potential lipid-related diagnostic biomarkers of acute myocardial infarction
Source: Aging (Albany NY). 2023 Feb 24;15(5):1394–411. doi: 10.18632/aging.204542 (PMC10042701; doi:10.18632/aging.204542)
Supplement: Supplementary Table 2 [file aging-15-204542-s003.pdf]

Supplementary Table 2. Key genes identified by LASSO and SVM-RFE.

Names	Total	GENES
LASSO SVM-RFE	4	ACSL1
		CH25H
		PLA2G12A
		GPCPD1
LASSO	15	ACSL1
		CH25H
		HSD11B1
		CYP1B1
		BMX
		PLA2G12A
		GPCPD1
		CROT
		ARV1
		TBXAS1
		GK3P
		LCLAT1
		ALOX5AP
		ORMDL2
		STARD4
SVM-RFE	4	GPCPD1
		ACSL1
		CH25H
		PLA2G12A
